# INVICTUS: Vitamin K antagonists remain the standard of care for rheumatic heart disease-associated atrial fibrillation

**DOI:** 10.21542/gcsp.2023.6

**Published:** 2023-01-30

**Authors:** Susy Kotit

**Affiliations:** Aswan Heart Centre (AHC), Aswan, Egypt

## Abstract

Introduction: Rheumatic heart disease (RHD) remains a major healthcare problem. Atrial fibrillation (AF) is the commonest sustained arrhythmia in RHD, leading to major complications and morbidity in a young population. Currently, anticoagulation with vitamin K antagonists (VKA) is the mainstay of therapy for the prevention of thromboembolic adverse events. However, effective use of VKA remains challenging, especially in developing countries, showing a need for alternatives. Novel oral anticoagulants (NOACs), including rivaroxaban, could form a safe and effective alternative to fulfil a major unmet need in RHD patients with AF. However, until recently, no data was available for the use rivaroxaban in patients with rheumatic heart disease associated AF.

Study and Results: The INVICTUS trial was conducted to assess efficacy and safety of once-daily rivaroxaban compared with a dose-adjusted VKA for the prevention of cardiovascular events in patients with RHD-associated AF. A total of 4531 patients (age: 50.5 ± 14.6 years) were followed for 3.1 ± 1.2 years in which 560/2292 patients in the rivaroxaban group and 446/2273 in the VKA group had a primary-outcome adverse event. The restricted mean survival time was 1599 days in the rivaroxaban group and 1675 days in the VKA group (difference, −76 days; 95% confidence interval [CI], −121 to −31; P <0.001). A higher incidence of death occurred in the rivaroxaban group than in the VKA group (restricted mean survival time, 1608 days vs. 1680 days; difference, −72 days; 95% CI, −117 to −28). No significant between-group difference in the rate of major bleeding was noted.

Lessons learned: The INVICTUS trial shows that Rivaroxaban is inferior to Vitamin K-antagonists in patients with RHD associated AF as VKA therapy led to a lower rate of ischemic and lower mortality due to vascular causes, without significantly increasing the rate of major bleeding. The results support current guidelines, which recommend vitamin K antagonist therapy for the prevention of stroke in patients with RHD associated AF.

## Introduction

Rheumatic heart disease (RHD) remains a major healthcare problem, affecting over 40 million people and causing significant morbidity and mortality on a global scale^1^. Atrial fibrillation (AF) is the commonest sustained arrhythmia in RHD, with a global prevalence of 32.8% in this patient group, increasing with the severity of valvular disease^2,3^. Individuals with AF due to RHD are young, and have significant cardiac morbidity^4–7^. Thus, the major complications of AF, which include peripheral thromboembolism, stroke, heart failure and premature death^8,9^ are of even bigger concern in these patients.

Currently, anticoagulation with vitamin K antagonists (VKA) is the mainstay of therapy for the prevention of thromboembolic adverse events^10^. However, regular coagulation monitoring and maintenance of the international normalized ratio required for the effective use of VKA, remains challenging^11–13^, especially in developing countries, showing the need for alternative therapies.

**Figure 1.**
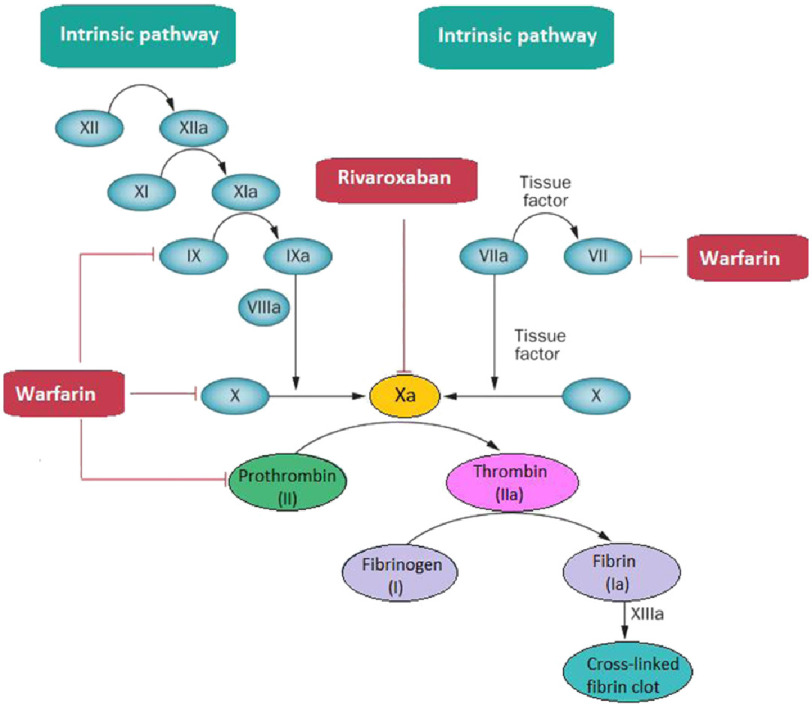
Coagulation cascade.

Rivaroxaban is a novel oral anticoagulant (NOAC) that falls under the direct factor Xa inhibitors^14^, a trypsin-like serine protease that plays a critical role in the blood coagulation cascade^15^ by linking the intrinsic and extrinsic pathways to the final common coagulation pathway, binding directly to factor Xa, thus preventing cleaving of prothrombin to thrombin^16^ (Figure 1).

Novel oral anticoagulants (NOACs) have been shown to be effective in the prevention of thrombosis in non-valvular AF^17–19^, and rivaroxaban was shown to be as effective as warfarin in the prevention of stroke or systemic embolism, with no increased risk of major bleeding^20,21^. As NOACs do not require monitoring and individualized dosing based on international normalized ratio of prothrombin time (INR), they could form a safe and effective alternative to VKA^22^ to fulfil a major unmet need in RHD patients with AF. However, until recently, no data was available for the use of rivaroxaban in patients with RHD-associated AF.

### The INVICTUS study

The INVestIgation of rheumatiC AF Treatment Using VKAs (INVICTUS-VKA) trial is the largest randomized trial conducted in RHD-associated AF and the first trial to assess anticoagulant therapy for stroke prevention in these patients^23^. INVICTUS was an international, multicentre, randomized, open-label, parallel group trial, conducted at 138 sites in 24 countries where RHD is endemic, mostly in Africa, Asia and Latin America.

The study was designed to assess efficacy and safety of once-daily rivaroxaban (Xarelto, Janssen/Bayer) 20 mg or 15 mg (based on renal function) compared with a dose-adjusted vitamin K antagonist for the prevention of cardiovascular events in patients with RHD-associated AF. Warfarin was the most-used vitamin K antagonist, (79–85% of the patients), and acenocoumarol was used in the others.

The primary efficacy outcome analysed was a composite of stroke, systemic embolism, myocardial infarction, or death from vascular or unknown causes. The primary safety outcome was the occurrence of major bleeding.

Patients enrolled in INVICTUS had an elevated stroke risk due to CHA2DS2 VASc score of 2 or higher (56%), a mitral valve area of 2 cm^2^ or less (82%), left atrial spontaneous echo contrast (11%) or left atrial thrombus (7%). The mean age was 50.5 years and nearly three-quarters of patients (72.3%) were women. These patients were randomly assigned to receive standard doses of rivaroxaban or dose-adjusted vitamin K antagonist.

Of 4565 enrolled patients, 4531 with RHD-associated AF were followed for a mean duration of 3.1 years. A primary efficacy endpoint event–which included stroke, systemic embolism, MI, or death from vascular or unknown causes–occurred in 560 out of 2292 patients assigned rivaroxaban (8.21% per year) and 446 out of 2273 assigned vitamin K antagonist therapy (6.49% per year) (proportional HR = 1.25; 95% CI [1.1–1.41]; *P* < .001).

Patients who received rivaroxaban therefore had a 25% increase in risk of a primary outcome. Most of this difference was because of the difference in mortality. Also, more patients assigned rivaroxaban experienced a stroke (90 vs. 65; proportional HR = 1.37; 95% CI, 1−1.89), primarily ischemic stroke.

Restricted mean survival time was 1599 days among those assigned rivaroxaban and 1675 among those assigned a vitamin K antagonist (difference, −76 days; 95% CI, −121 to −31; *P* < .001 for superiority). Incidence of death was higher in the rivaroxaban group compared with the vitamin K antagonist group (restricted mean survival time: 1,608 days vs. 1680 days; difference, –72 days; 95% CI, –117 to –28), showing a restricted mean survival time difference of 72 days over a period of 3.1 years of follow-up. The difference in mortality was entirely due to lower rates of sudden cardiac death and death due to mechanical pump failure in the vitamin K antagonist group.

There was no significant difference in the rate of International Society of Thrombosis and Hemostasis major bleeding between the two groups (rivaroxaban, 40 patients (0.67% per year); vitamin K antagonists, 56 patients (0.83% per year); proportional HR = 0.76; 95% CI [0.51–1.15]; P = 0.18). However, the rate of fatal bleeding was lower with vitamin K antagonist therapy (4 vs. 15 patients; proportional HR = 0.29; 95% CI [0.1–0.88]).

No between-groups differences were observed in the rate of hospitalizations for heart failure or indication for valvular procedure.

Permanent discontinuation of the study drug was more common in the Rivaroxaban group (about 23%), with the most common reasons cited as hospitalization for valve surgery and patient decision.

## Discussion

Rivaroxaban has been successful in the prevention of stroke in patients with atrial fibrillation not related to valvular disease.^17–19^ However, in a predominantly female, younger population with RHD-associated AF, vitamin K antagonist therapy led to a lower rate of a composite of cardiovascular events or death than rivaroxaban therapy, without a higher rate of bleeding^23^.

One of the possible explanations given by the investigators were the more frequent follow-up visits for INR monitoring in the VKA group, which might have led to better care and fewer strokes and deaths and lower adherence to rivaroxaban therapy. However, the results of the INVICTUS support that adjusted-dose vitamin K antagonists should remain the standard of care and stroke prevention for RHD-associated AF, but further in-depth research is warranted in order to analyze the differences found.

AF associated with RHD remains a challenging health problem with high morbidity and mortality in a significantly young population. Although vitamin K antagonists have been shown to be more effective in reducing the rate of cardiovascular events and death, due to many dietary and pharmacologic interactions, bleeding risk, narrow therapeutic index, regular need for blood sampling and individualized dosing based on INR, vitamin K antagonist therapy is difficult to administer and control.

### Lessons learned

• The INVICTUS trial shows that rivaroxaban is inferior to vitamin K-antagonists in patients with RHD-associated AF.
• The trial showed that compared with rivaroxaban, vitamin K antagonist therapy led to a lower rate of ischemic and lower mortality due to vascular causes, without significantly increasing the rate of major bleeding.
• The results of this trial support current guidelines, which recommend vitamin K antagonist therapy for the prevention of stroke in patients with RHD-associated AF, with frequent control of INR.
